# Connectivity modelling identifies sources and sinks of coral recruitment within reef clusters

**DOI:** 10.1038/s41598-024-64388-8

**Published:** 2024-06-12

**Authors:** Chinenye J. Ani, Vanessa Haller-Bull, James P. Gilmour, Barbara J. Robson

**Affiliations:** 1 Australian Institute of Marine Science, PMB3 Townsville, Townsville, QLD 4810 Australia; 2 Australian Institute of Marine Science, Indian Ocean Marine Research Centre, The University of Western Australia, Crawley, Western Australia Australia; 3 AIMS@JCU, Australian Institute of Marine Science, College of Science and Engineering, James Cook University, Townsville, QLD 4811 Australia; 4 Oceans Institute, The University of Western Australia, Crawley, Western Australia Australia

**Keywords:** Environmental impact, Climate change, Physical oceanography

## Abstract

Connectivity aids the recovery of populations following disturbances, such as coral bleaching and tropical cyclones. Coral larval connectivity is a function of physical connectivity and larval behaviour. In this study, we used OceanParcels, a particle tracking simulator, with 2D and 3D velocity outputs from a high resolution hydrodynamic-biogeochemical marine model (RECOM) to simulate the dispersal and settlement of larvae from broadcast spawning *Acropora* corals in the Moore Reef cluster, northern Great Barrier Reef, following the annual spawning events in 2015, 2016 and 2017. 3D velocity simulations showed 19.40–68.80% more links and sinks than those of 2D simulations. Although the patterns of connectivity among sites vary over days and years, coral larvae consistently dispersed from east to west in the cluster domain, with some sites consistently acting as sources or sinks for local larval recruitment. Results can inform coral reef intervention plans for climate change, such as the design of marine protected areas and the deployment of proposed interventions within reef clusters. For example, the wider benefits of interventions (e.g., deployment of heat adapted corals) may be optimised when deployed at locations that are a source of larvae to others within comparable habitats across the reef cluster.

## Introduction

The Great Barrier Reef (GBR) is the largest coral reef system in the world. Located along the North-eastern coast of Australia, the GBR covers an area of approximately 344,000 km^2^ and supports an abundance of marine life, including about 600 coral species and 1600 fish species^1,2^. Although the GBR is one of the most managed marine ecosystems in the world, it is under pressure from the impacts of climate change, natural disturbances and other human activities^3–6^. The deteriorating condition of the GBR has increased the urgency of understanding how this unique ecosystem will respond to future environmental changes.

Ocean currents in the GBR transport marine organisms from one site to another, allowing exchange between populations. For organisms with a pelagic larval stage, connectivity among sites aids recovery of populations following disturbances, such as coral bleaching and tropical cyclones^7^. Connectivity also allows genetic mixing between organisms from different populations^8,9^ and the spread of organisms to new reefs, facilitating range expansion^10^. Coral larval connectivity is a function of physical connectivity (ocean currents at the time of larval production) and larval production and behaviour (spawning times, minimum and maximum competency periods, mortality rates, larval behaviour and settlement)^11,12^. Depending on current speeds and larval ecology, dispersal in the GBR can occur over scales ranging from metres to hundreds of kilometres. The spatial scales over which coral populations are connected is determined by the net effect of these processes^13^, with very different implications of dispersal distances over demographic, ecological and evolutionary time scales. Therefore, quantifying the dispersal of coral larvae within and between reef systems is critical for identifying sources and sinks of recruitment, and for designing marine protected areas and the deployment of active interventions.

It is impossible to track coral eggs, embryos and larvae, given their small size, the complexity of their biology and behaviour over days to weeks of dispersal, and their interactions with changing conditions within a vast ocean^14,15^. Consequently, different methods are used to infer dispersal distances and connectivity in corals, such as stock-recruitment relationships^16,17^, coral population genetics^18,19^ and biophysical models^20,21^. All of these methods have limitations, either logistically or in the capacity to detect patterns of connectivity over demographic, ecological or evolutionary time steps. Of all the methods, biophysical models are most commonly used to assess demographic connectivity, by exploring dispersal among populations within reef clusters over years to decades^11,20,22–26^. Biophysical models explicitly simulate the hydrodynamics in the region of interest, larval production, dispersal and behaviour prior to settlement.

To best inform management of coral reefs, biophysical models should match the spatial and temporal scales at which management actions, such as reef restoration, are to be applied. Models therefore need to resolve currents down to the scale of hundreds of meters, because local-scale currents near reefs and islands impact large-scale circulation patterns around continuous reef systems^27^ and increase local retention of larvae in and around their natal reefs^21,28,29^. Tidal currents also generate fine-scale eddies on and around reefs, which trap larvae by repeatedly transporting them over or around reefs over several days^28,30–33^. This re-circulating process can reduce the transport of larvae away from the reef cluster, reducing long distance dispersal^21^. In the GBR, a reef cluster consists of all patch reefs enclosed in a rectangular domain of approximately 625 km^2^ and contains important ecological, environmental and socio-economic dimensions of the GBR as a socio-ecological system. Therefore, neglecting local-scale currents likely overestimates larval dispersal distances between adjacent coral reef atolls and underestimates within-reef connectivity^21^.

Several studies have investigated GBR connectivity using low resolution biophysical models. eReefs hydrodynamic model configured at 4km-resolution and Lagrangian particle tracking simulators have been used to infer a high degree of mixing across the GBR in certain reef fish^26^; simulate the dispersal of crown-of-thorns starfish (CoTS) larvae in the GBR and identify important reefs where CoTS outbreak originate^23^; estimate that about 100 reefs in the GBR have the potential to recover from disturbances because they are highly connected, have low exposure to disturbances and are not sources of CoTS outbreaks^34^; and show that split spawning events may increase the reliability of coral larval supply^35^.

In addition to the resolution of hydrodynamic models, the parameters used to estimate the distribution and larval ecology of target organisms fundamentally affect estimates of connectivity across space and time. For example, fine-scale hydrodynamic models have explored larval connectivity between shallow and deep reefs and the effects of climate change on larval duration (i.e., period during which corals exist as larvae) and mortality^20,24^. However, very different connectivity estimates may be obtained when applying even fine-scale hydrodynamic models to coral species with different distributions and modes of reproduction (e.g., brooding or spawning corals), or choosing different demographic traits (e.g., minimum competency period) and outcomes of environmental variation, such as climate change. Biological parameters for coral larvae are usually inferred from laboratory experiments, with sparse data^36^ available for natural rates of larval development, behaviour and mortality. This lack of biological data remains a major impediment to producing realistic estimates of connectivity among coral populations, with the reliability of estimates rapidly decreasing with increasing spatial and temporal scales of modelling.

**Figure 1:**
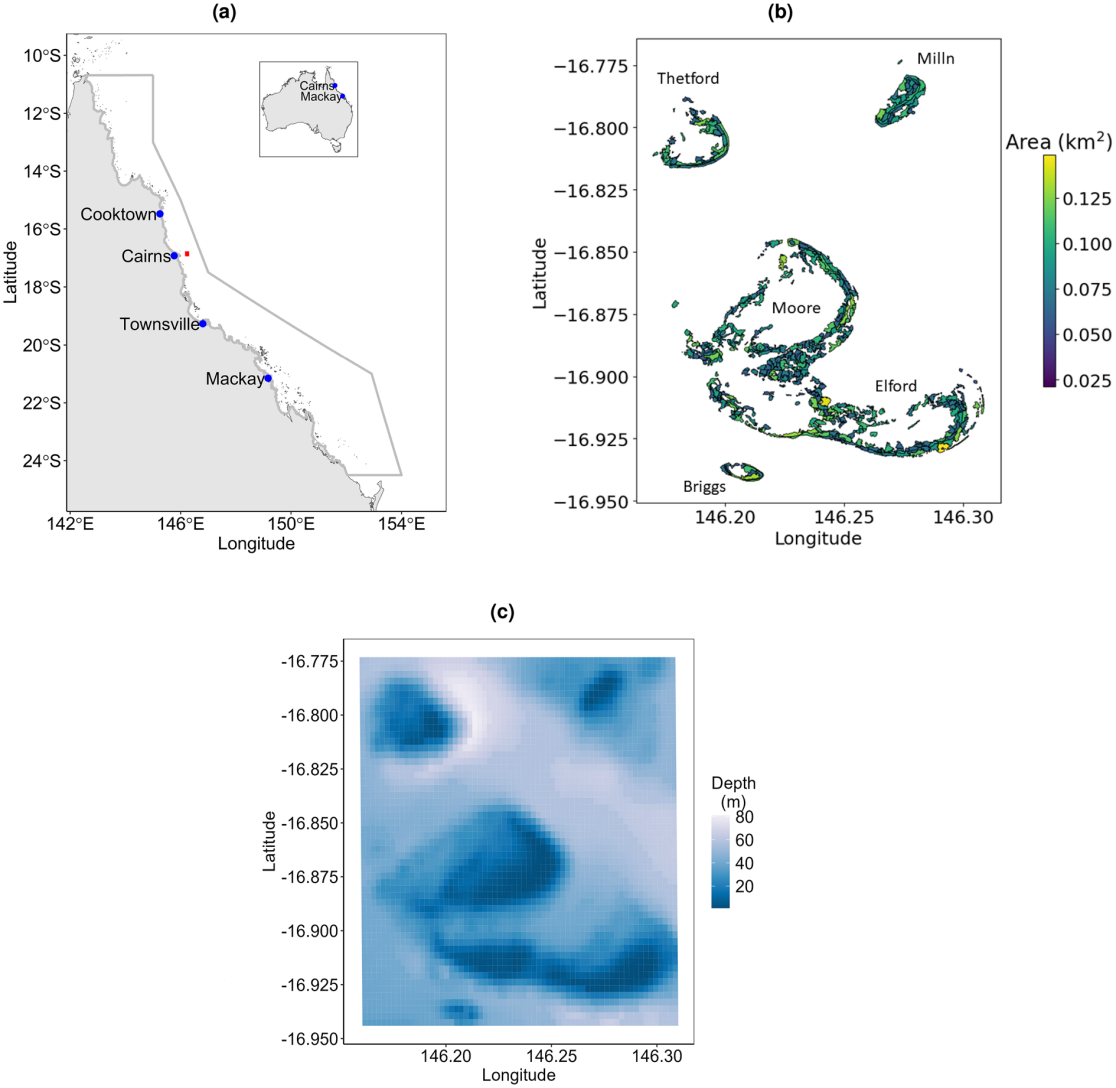
Map showing the location of the Moore Reef cluster (red) in the Great Barrier Reef Marine Park boundary (grey line) (**a**). Map showing the spatial polygons and reefs within the Moore Reef Cluster domain (**b**). The colour bar labelled ‘Area’ represents the area of the spatial polygons. Map showing the bathymetry of the Moore Reef Cluster domain (**c**).

To date, limited fine-scale ($\sim$ 200 m) coral larval connectivity modelling has been conducted on the GBR and few models have used 3D velocities to resolve vertical flows. 2D- and 3D-velocity predictions of particle retention rates in GBR shelf waters have been reported to be similar, except for particles located at preferred depth layers due to vertical changes in horizontal currents^32^. In this study, we used a biophysical modelling approach to simulate the dispersal and settlement of larvae from broadcast spawning *Acropora* corals in the Moore Reef cluster (Fig. 1b) of reefs following the annual mass-spawning in 2015 and 2016, and split-spawning in 2017. Larval dispersal was simulated using OceanParcels^37^, a Lagrangian particle tracking simulator, with hourly 2D and 3D velocity outputs from a 200–250 m resolution hydrodynamic-biogeochemical marine model (RECOM—Relocatable Coastal Model https://research.csiro.au/ereefs/models/models-about/recom/) nested within a 1 km grid resolution hydrodynamic model of the Great Barrier Reef Marine Park (https://research.csiro.au/ereefs/models/models-about/models-hydrodynamics/). Particles were randomly released within 334 spatial polygons representing reef sites within the cluster domain from 8:00 pm to 11:00 pm during three spawning nights across the spawning years considered.

**Figure 2:**
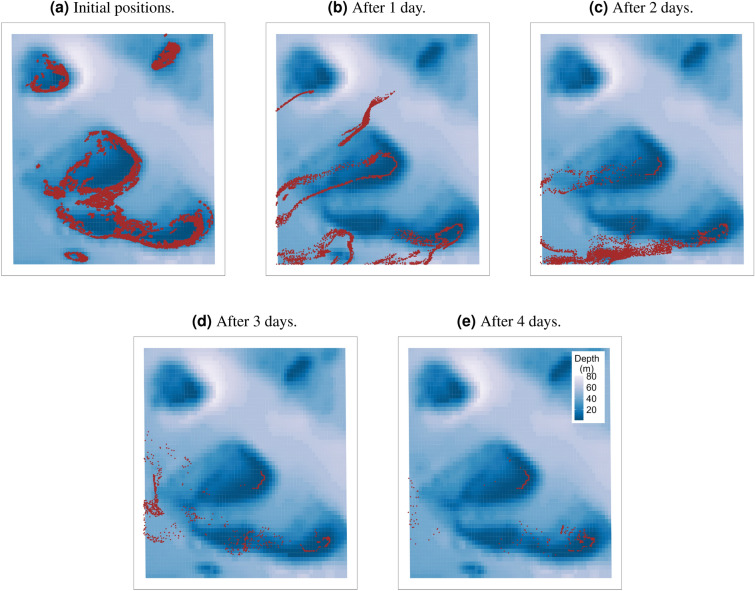
Snapshots showing the dispersal of *Acropora* coral larvae within the Moore Reef cluster for spawning day 3 in 2015. 2D surface velocity fields were used to simulate larval dispersal. Brown dots represent particles and the colour bar represents the bathymetry of the cluster. The initial positions of the particles are shown in a, whilst particle positions after 1, 2, 3 and 4 days are shown in b, c, d and e.

**Figure 3:**
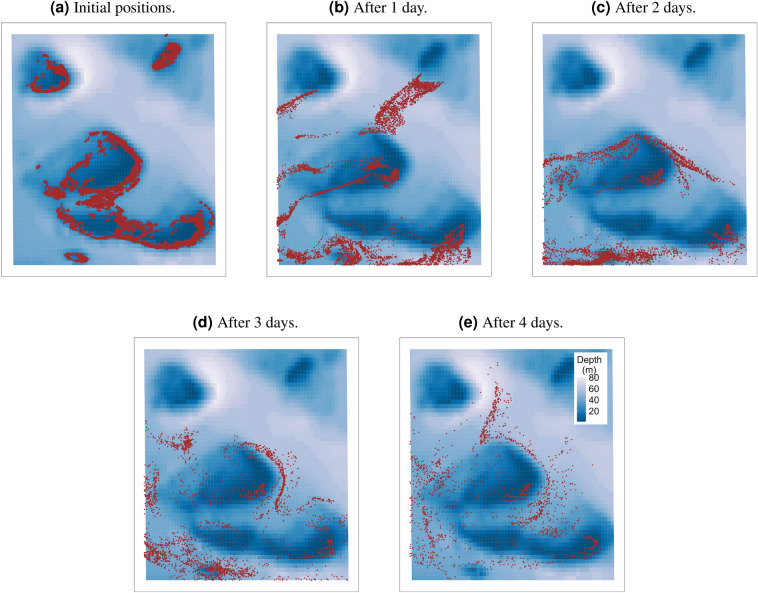
Snapshots showing the dispersal of *Acropora* coral larvae within the Moore Reef cluster for spawning day 3 in 2015. 3D velocity fields were used to simulate larval dispersal. Brown dots represent particles and the colour bar represents the bathymetry of the cluster. The initial positions of the particles are shown in a, whilst particle positions after 1, 2, 3 and 4 days are shown in b, c, d and e.

Connectivity can vary dramatically among years according to the times of larval production and the corresponding variation in environmental conditions, such as local tides, winds and currents. Indeed, the timing of storms or the passing of tropical cyclones can often change the patterns of connectivity among populations, as can the distribution and abundance of coral species across a cluster of reefs^21^. Consequently, management strategies require an understanding of which sites are consistently important as sources or sinks (i.e., larval receiving sites) within a cluster of reefs across multiple spawning periods. Here we identify consistent source and sink locations for coral larvae within the Moore Reef cluster, northern GBR, off Cairns (Fig. 1) over four spawning periods and highlight the need to consider different environmental conditions when assessing connectivity (Figs. 2 and 3).

## Results

### Model sensitivity to the dimension of velocity fields

**Figure 4:**
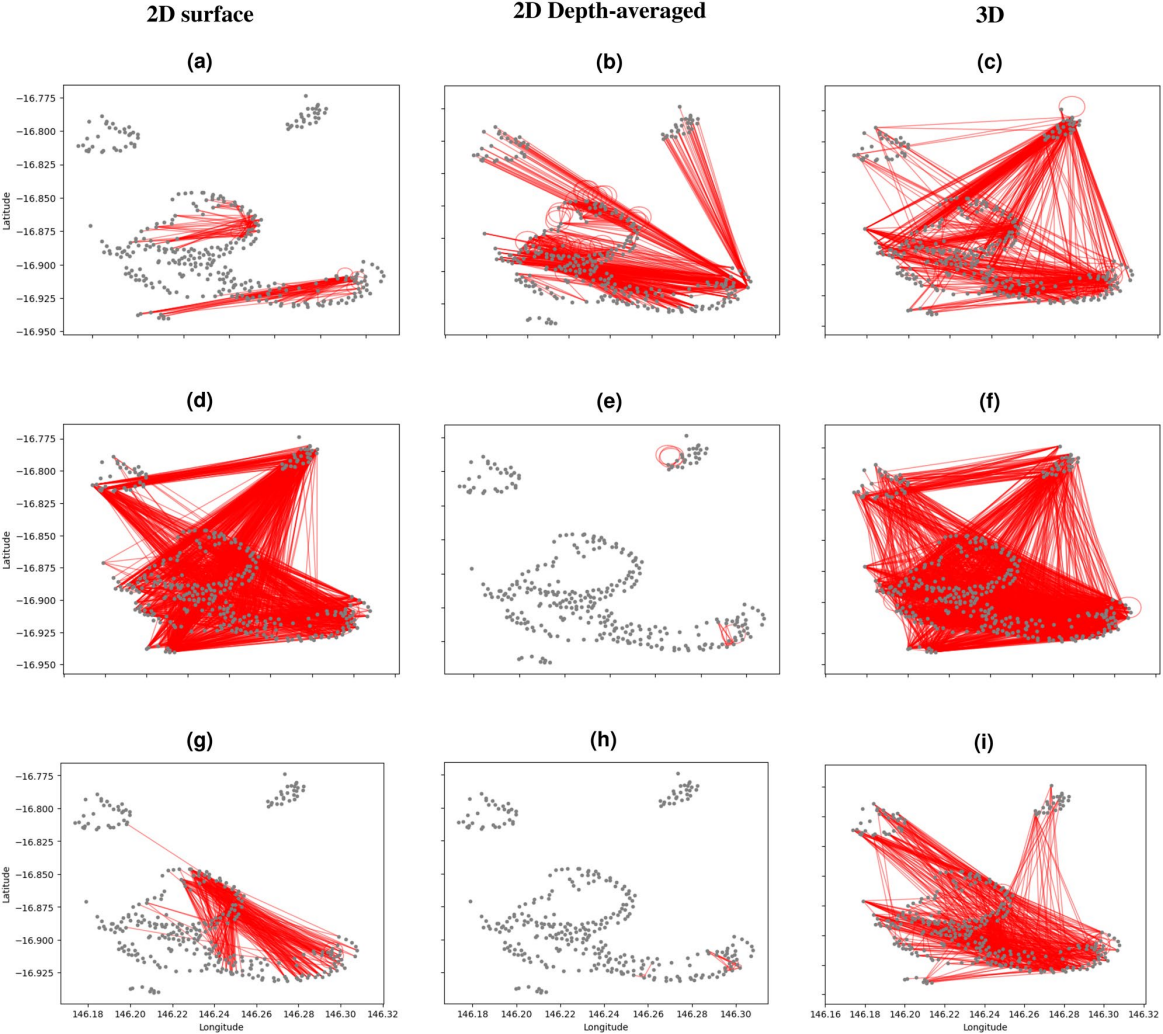
Connectivity networks for *Acropora* coral in the Moore Reef cluster of the Great Barrier Reef for spawning day 3 across all spawning years. 2015 spawning event (a, b and c), 2016 spawning event (d, e and f), 2017 first spawning event (g, h and i). For subplots a, d and g (left plots), 2D surface velocity fields were used, for subplots b, e and h (middle plots), 2D depth-averaged velocity fields were used whereas 3D velocity fields were used for subplots c, f and i (right plots). Nodes (points) represent the centroids of reef site polygons, lines or arcs represent larval exchange between reef sites. A link is shown when larvae move from one node to settle on another node (straight line) or remain at the source node (cyclic line).

To determine the influence of the dimension of velocity fields on connectivity estimates, we compare results from model simulations forced with 2D surface velocities (Fig. 4a,d,g), 2D depth-averaged velocities (Fig. 4b,e,h) and 3D velocities (Fig. 4c,f,i) for three spawning days in 2015, 2016 and 2017 (first spawning event). To isolate the impacts of the dimension of velocity fields, coral larval mortality was not considered in this sensitivity analysis. The percentage of total released coral larvae that settle on reef sites within the cluster domain and the percentage of reef sites that act as settling sites are shown in Table 1.

Over the dispersal period, 3D velocity simulations showed showed 19.40–68.80% more links and sinks than those of 2D simulations (Table 1 and Fig. 4). This is because larvae are more likely to sink to slower-moving waters within the reef lagoon below the surface^32^ (Fig. 3, Supplementary Figs. S5 and S8). For the 2D-surface-velocity simulations, larvae were always at the surface throughout the dispersion period where they are easily swept off by wind-driven currents^32^ (Fig. 2, Supplementary Figs. S3 and S6). For the depth-averaged velocity simulations, most larvae were dispersed out of the cluster domain before the start of the competency period during 2016 and 2017 spawning events resulting in very low connectivity estimates (Table 1 and Supplementary Figs. S4 and S7). This could be as a result of not considering the vertical movement of larvae during dispersal, which can slow down the horizontal movement of larvae. The percentage of larvae that settled within the cluster domain during the dispersal period varied in the three scenarios (Table 1). For the surface, depth-averaged and 3D simulations, the connectivity networks for the three years were different; 2016 and 2017 depth-averaged simulations exhibited the lowest number of connections whereas the highest number of connections occurred in 2016 surface and 3D simulations (Fig. 4).

The use of 2D velocity fields may be sufficient in (near) barotropic conditions, which occur in well-mixed waters usually found in shallow shelf waters or surface waters. However, currents in the Moore Reef cluster are most likely affected by baroclinic processes due to the offshore location (Fig. 1a) and bathymetry (Fig. 1c) of the cluster. Also, Supplementary Fig. S1 shows that surface, 2D depth-averaged and bottom velocities within the cluster are different. Therefore, it is better to use 3D velocities as they resolve upwelling and downwelling effects and consider the effects of the vertical movement of larvae. Hence, the use of 2D velocity fields might result in an under-estimation of the cluster’s connectivity.

**Table 1:** Moore Reef cluster, northern Great Barrier Reef. Percentage of total released coral larvae that settled at reef sites within the cluster and percentage of sink sites during the 2015, 2016 and 2017 (first) spawning events for 2D and 3D velocities.

	Spawning year	Day	Surface velocities (2D)	Depth-averaged velocities (2D)	3D velocities
Percentage of larvae that settle	2015	1	5.20	42.80	4.60
		2	2.90	42.80	1.80
		3	5.20	42.50	5.30
	2016	1	2.30	1.50	20.10
		2	3.80	1.50	12.90
		3	14.90	1.50	13.40
	2017	1	1.00	1.50	2.10
		2	2.90	1.50	2.40
		3	5.90	1.50	3.90
Percentage of sites that act as settling sites	2015	1	18.00	48.10	43.10
		2	15.60	56.10	35.00
		3	18.00	57.00	52.10
	2016	1	17.70	0.60	86.50
		2	25.70	0.60	86.20
		3	53.30	0.60	85.90
	2017	1	7.80	1.20	44.90
		2	9.60	1.20	45.20
		3	10.20	1.20	55.70

**Figure 5:**
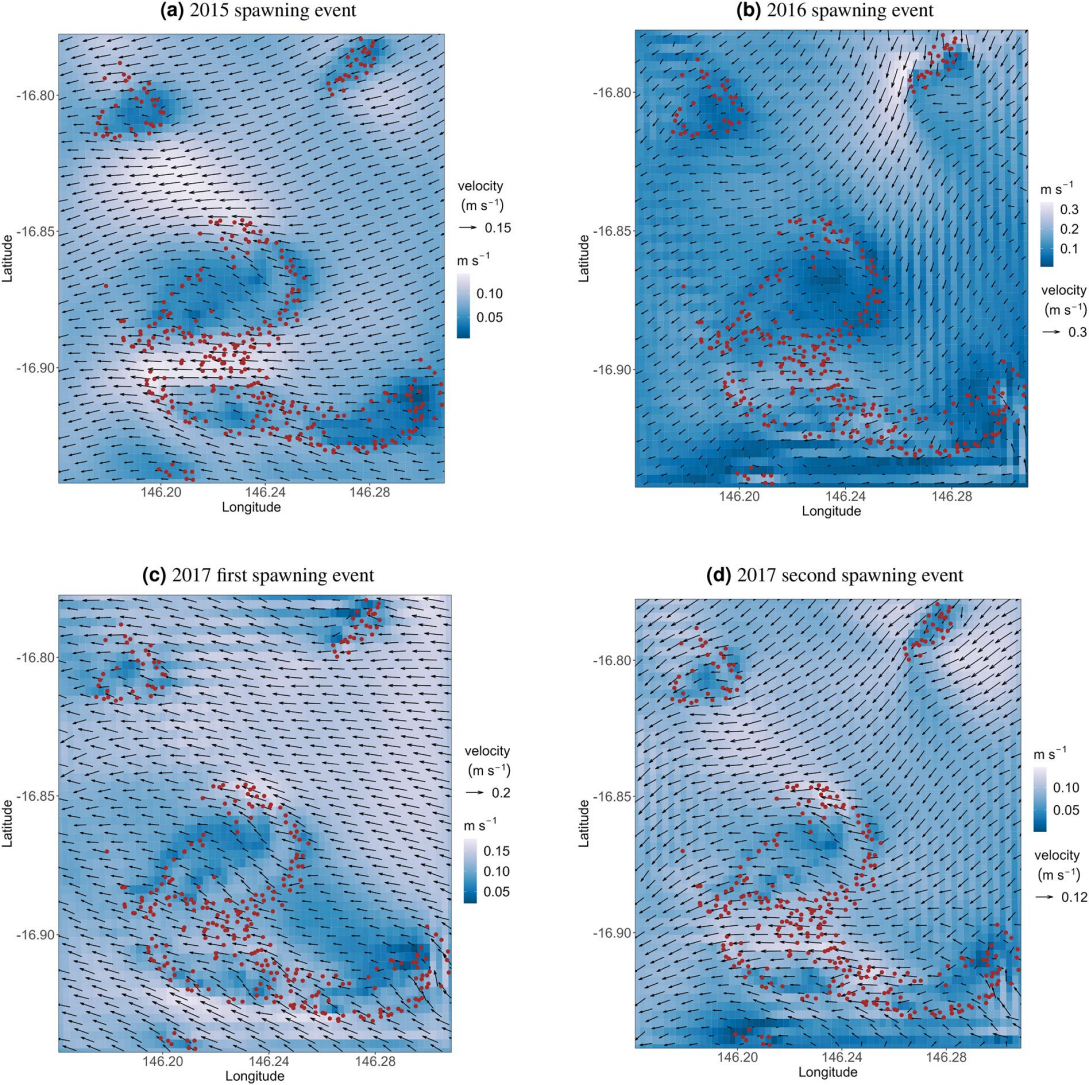
Time-averaged surface flow patterns during the larval dispersal period for all spawning events. Brown dots represent the centroids of reef site polygons.

### Consistent source and settling (sink) sites

3D velocities and 0.40 d^-1^ mortality rate were used for the connectivity model simulations for spawning nights during each of the three years (2015–2017). The 0.40 d^-1^ mortality rate is our best estimate of mortality based on analysis from published *in situ* and laboratory data on larval mortality after coral spawning^36,38,39^.

**Figure 6:**
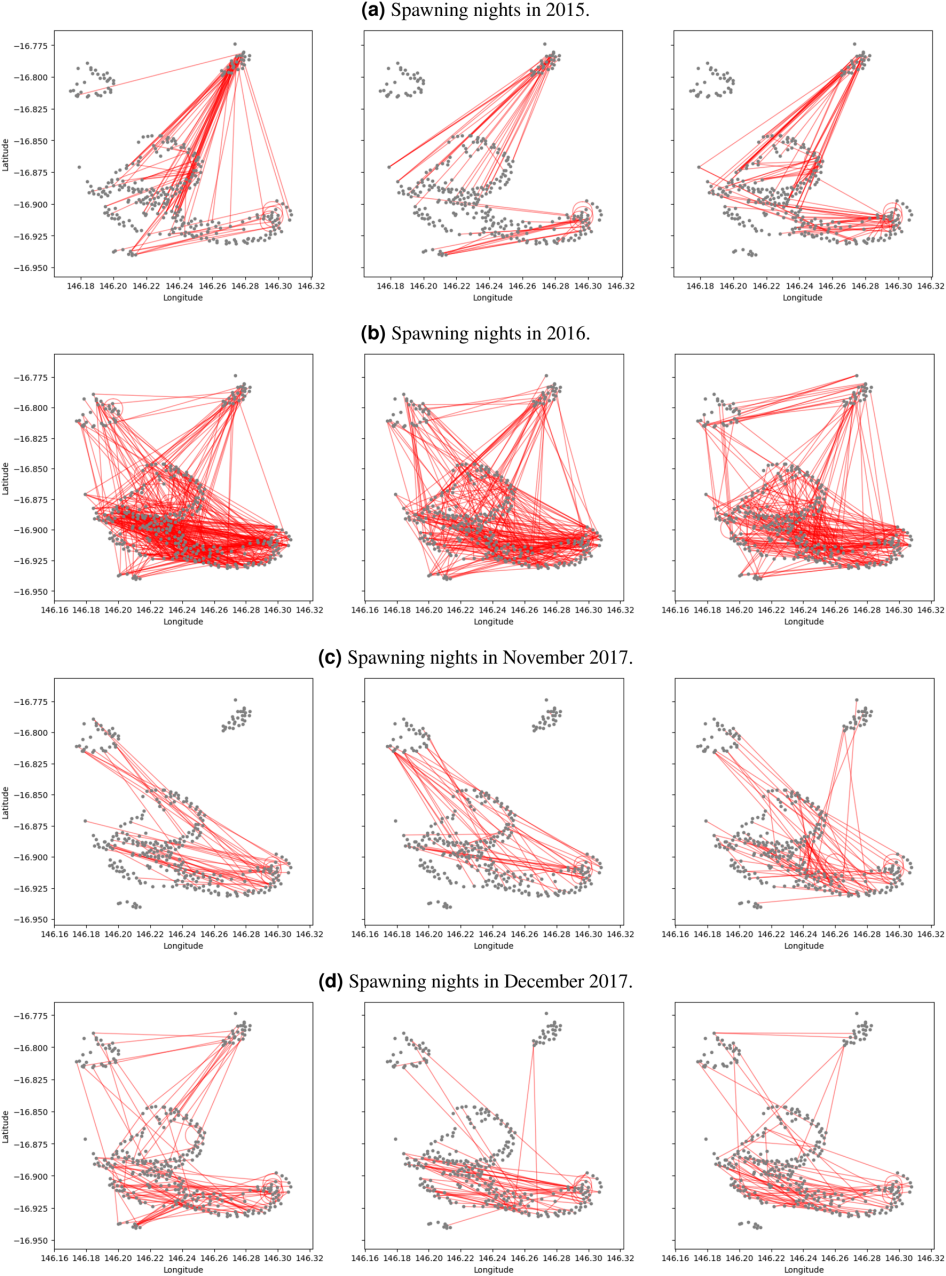
Connectivity networks for *Acropora* coral in the Moore Reef cluster of the Great Barrier Reef for three nights of spawning in 2015 and 2016, and six nights in 2017. From left to right: spawning nights 1, 2 and 3. Nodes (points) represent the centroids of reef site polygons and red lines or arcs represent larval exchange between reef sites. A link is shown when larvae move from one node to settle on another node (straight line) or remain at the source node (cyclic line).

Time-averaged flow patterns during larval dispersal is generally from offshore to onshore across all spawning events (Fig. 5). Larval exchange among sites (polygons) varied from 0.30 to 2.70% during the different nights and years of spawning, but with highest exchange among sites occurring during 2016 (Fig. 6). Although the patterns of connectivity among sites vary over days and years (Fig. 6), coral larvae consistently dispersed from east to west in the cluster domain, with some sites consistently acting as sources (i.e., release sites) or sinks (i.e., settling sites) for local larval recruitment.

**Figure 7:**
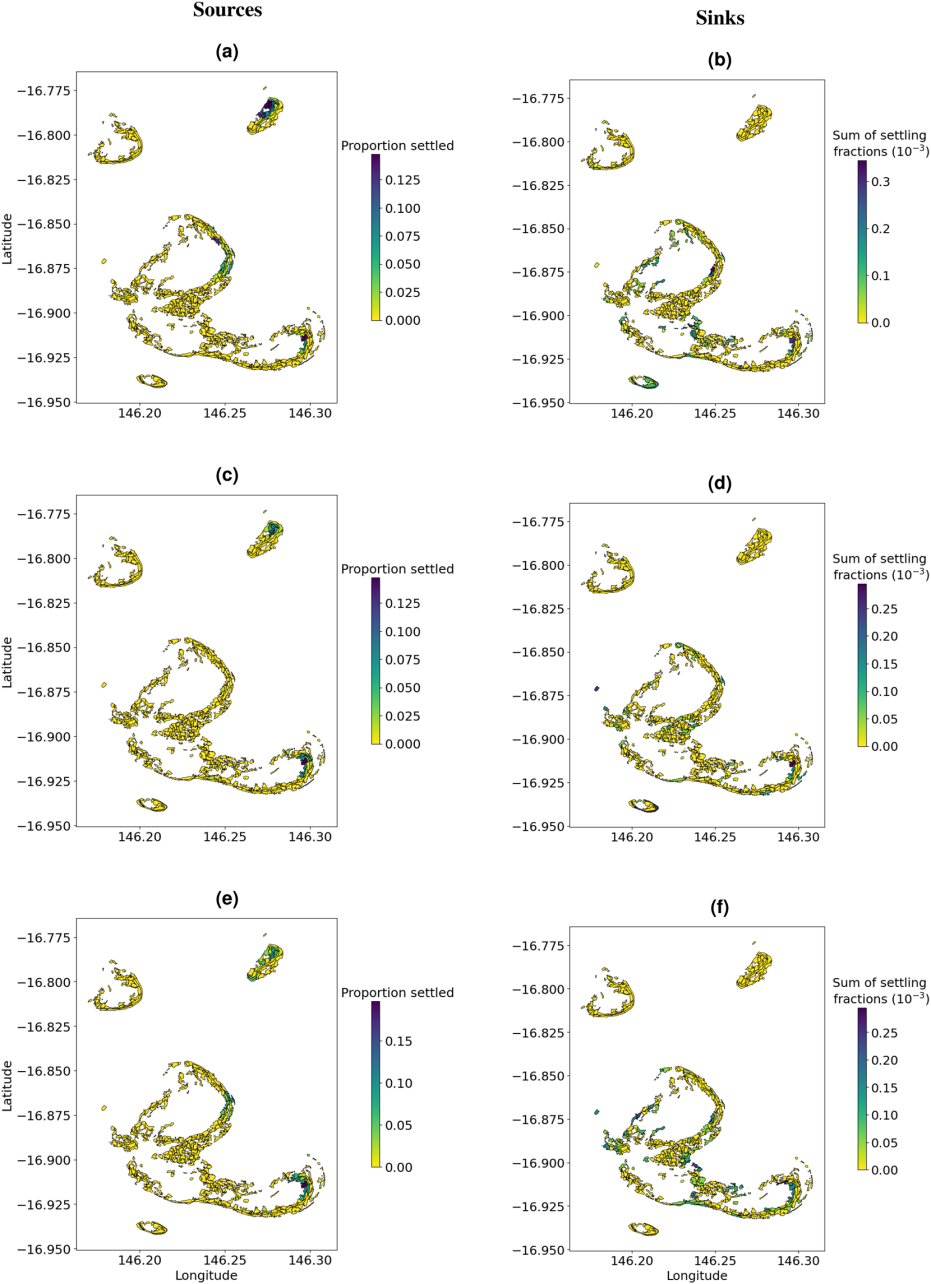
Map showing reef sites that act as local sources (a, c and e) and sinks (b, d and f) during the 2015 annual mass spawning. Spawning night 1 (a and b), spawning night 2 (c and d), spawning night 3 (e and f). The colour bar labelled ‘Proportion settled’ corresponds to the proportion of released larvae from each reef site that settle on one or more other sites (a, c and e). The colour bar labelled ‘Sum of settling fractions’ corresponds to the sum of the proportion of the total released larvae from all sites that settle on a particular site (b, d and f).

During 2015, sources were located on Milln, Moore and Elford Reefs, whereas settling sites were mostly located in the southern part of the cluster (Fig. 7). During the 2016 spawning event, all reefs had source and settling sites (Supplementary Fig. S9). During the first split-spawning event in 2017, sources were located on Moore and Elford Reefs, whereas settling sites were on Thetford, Moore, Elford and Milln Reefs (Supplementary Fig. S10). During the second spawning event in 2017, sources were located on Moore, Elford and Milln Reefs, whereas settling sites were on Thetford, Moore, Elford and Briggs Reefs (Supplementary Fig. S11).

**Figure 8:**
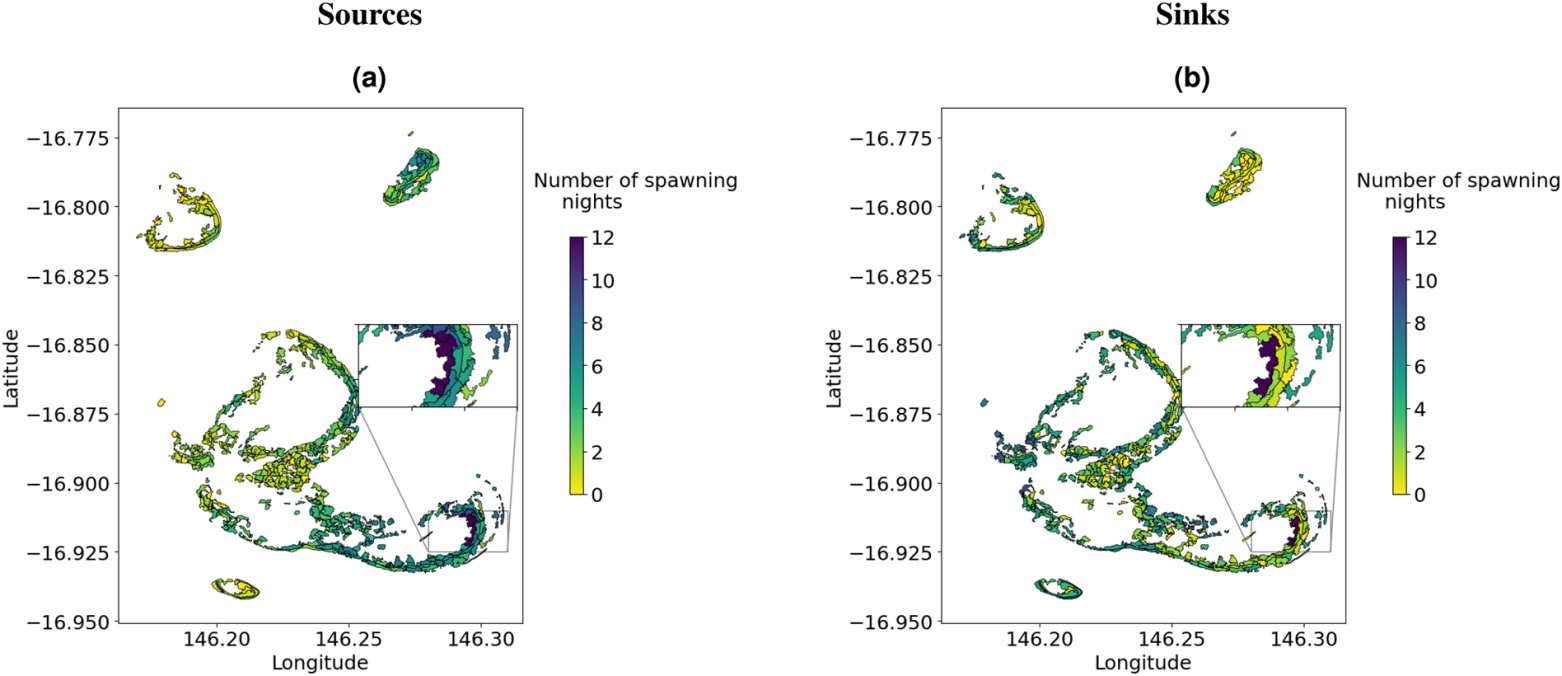
Map showing the number of spawning nights reef sites act as local sources (**a**) and sinks (**b**) during the spawning events in 2015, 2016 and 2017. The zoomed-in plots show reef sites that consistently act as local sources (**a**) and sinks (**b**) across all spawning nights.

All the reefs had sites that acted as local sources and sinks during one or more spawning nights (Fig. 8a,b). The zoomed-in plots in Fig. 8a,b show key source and settling sites that were consistent over nights and years of spawning. In particular, sites located at the easternmost part of Elford Reef act as sources and sinks across all simulated spawning events. Self-recruiting sites during one or more spawning nights are shown in (Supplementary Fig. S12).

## Discussion

In this study, we used a fine-scale (200–250 m) hydrodynamic model and a Lagrangian particle tracking tool to infer coral larval dispersal and settlement in the Moore Reef cluster. The use of 3D velocity fields nested within 1 km 3D velocity fields at hourly time steps allowed the resolution of local-scale currents and flows between reefs, which greatly influence larval dispersal^27^. However, there are smaller unresolved scales that affect larval dispersal that can be captured by explicit diffusion and can improve ability to estimate connectivity.

The modelling approach used in this study which includes nesting 3D velocities and randomly releasing larvae within each of the spatial polygons allowed the estimation of fine-scale connectivity. Our model outputs reflect the influence tidal circulation, waves, ocean currents and the dimension of velocity fields can have on coral larval dispersal and connectivity. The connectivity networks and estimates for surface, depth-averaged and 3D velocities are different, with 3D velocity simulations having the highest degree of connectivity. This is because 3D velocities best resolve the hydrodynamics of the Moore Reef cluster since the water column is not well mixed due to the offshore location and bathymetry of the cluster (Fig. 1). Oscillating tidal currents are mostly cross-shelf in the GBR and time-averaged flow patterns in the cluster domain during larval dispersal have been shown to be from east to west (i.e., offshore to onshore) (Fig. 5).

Our modelling results show variability in larval connectivity, among consecutive nights of spawning during a single year and among years. Across all spawning events, links between sites (populations) appeared and disappeared (Fig. 6). The change in interannual within-reef connectivity is likely caused by the temporal variability in tides during the spawning events and passing storms and cycles, which have been shown to alter waves and therefore within-reef connectivity^21^. Larval dispersal is highly reliant on hydrodynamics during spawning events^40^ and changes in oceanographic conditions have been reported to cause the highly stochastic nature of marine ecosystems connectivity^41,42^.

Biophysical models can provide information on the resilience or vulnerability of coral reefs to disturbances based on the extent by which reefs are connected to potential sources of larvae. Our model results show that some locations were consistently important across different spawning years and environmental conditions (Fig. 8a,b), while other locations were only important in some years. Although only about four sites consistently acted as sources and sinks during the simulated spawning events, Fig. 8a,b show a high degree of connectivity across all the spawning events.

The modelling approach used in this study provides estimates of hydrodynamic movements within the reef clusters around the times of larval dispersal, but the three years considered do not provide sufficient inter-annual current variability to build robust connectivity indicators. In addition, our connectivity estimates are biased by a lack of rigorous biological data. The modes of reproduction (e.g., hermaphroditic broadcast spawners) and times for mass spawning and larvae production are well documented for many *Acropora* species on many parts of the GBR^43,44^, but we still know very little about other aspects of coral larval ecology or how variation in these parameter estimates may affect connectivity estimates. For example, larval competency periods and survival rates are based almost entirely on laboratory studies, while larval movement within the water column and their capacity to respond to different environmental cues (eg., irradiance) within different flow regimes on the reef are unknown (e.g.,^36,39,45^). Given the difficulty in quantifying many of these parameters, there is need for sensitivity analyses that include presumed variation in spawning windows, competency periods, mortality rates, vertical and horizontal movements through stages from spawning to larval settlement.

The sensitivity of the biophysical model results to varying estimates of these biological parameters can help address these gaps by identifying which parameters are most influential and how connectivity matrices vary with different estimates of parameter values, while also highlighting research priorities. Since Figueiredo et al.^46^ have established that increased mortality rate reduces connectivity, for sensitivity analysis, we will need to explore a range of mortalities closer to our best estimate of true mortality^39^. Ideally, this should be considered in combination with changes to other model parameters, such as release times and number of particles released at each site, but there is limited biological data for model parameterisation. Thus, there is need for more *in situ* data on natural larval mortality in the GBR for more reliable within-reef coral larval connectivity estimates.

Furthermore, the biophysical model used in this study does not simulate some important larval behaviour such as time required to settle, explore the substrata and metamorphose when over a reef, which in turn varies with current speeds and structural complexity of the reef community. In addition, because coral connectivity in the GBR is a function of changing atmospheric-oceanic conditions, there is a need to consider connectivity patterns over multiple years. Finally, our estimates of connectivity are for a generic, mass spawning hermaphroditic coral with neutrally buoyant gametes, whereas many other species of corals have very different reproductive traits, such as brooding rather than broadcast spawning, different times of spawning or release, colonies of separate sexes and/or gametes that are neutrally or negatively buoyant^44,47,48^. Revising and incorporating these factors in future biophysical models will improve connectivity predictions for communities of corals in the GBR.

## Implications for management

From a management perspective, our model results highlight the importance of assessing connectivity across several environmental conditions to help provide reliable connectivity model predictions for effective coral conservation in the GBR.

Our findings identify reef locations with high potential for interventions within reef clusters in the GBR. Reefs that receive larvae from other reefs are sinks and are more persistent and resilient to disturbances. They can recover quicker from local or global stressors because they have a stable source of larval recruits. Therefore, sinks may be more resilient to local and global stressors such as coral bleaching and tropical cyclones. Reefs that provide larvae to other reefs act as local sources and could be important for maintaining coral populations and for the dispersal of offspring (e.g., heat adapted) generated by restoration efforts.

Our model results show that local-scale (within-reef) coral larvae connectivity patterns can modify reef population in the GBR, and is therefore critical for developing efficient spatial management and coral conservation plans for improving the resilience of the GBR to climate change and other future pressures.

## Methods

### Study site

The Moore Reef cluster spans approximately 20 km and comprises Moore Reef, Milln Reef, Thetford Reef, Elford Reef and Briggs Reef (Fig. 1b). This region experiences high tourist activity, particularly on Moore Reef which consists of national parks and conservation zones. During austral spring and summer (2010 – 2019), the lagoonal branch of the East Australian Current extending from the central to southern GBR is the dominant current in this region and shelf current velocities near the surface were driven onshore by the wind^49^.

The cluster consists of 334 spatial polygons which are used to represent different reef sites within the cluster (Fig. 1). Geomorphic maps from Roelfsema et al.^50^ were used to establish the boundaries of the spatial polygons. From these geomorphic maps, only the habitats and environmental conditions suitable for *Acropora* corals were included, namely the Reef Slope, Reef Crest, Sheltered Slope and Outer Reef Flat. The creation of the polygons is a multistep process that starts with the separation of the area into the four habitat zones. Within each of these habitats, polygons should include hexagons on the geomorphic map that are close together with a target area of around 62,500 m^2^ (RECOM grid size) and with similar depth. To achieve this, a step-wise spanning tree approach was applied. The first step involved the use of Delaunay triangulation to identify hexagons that could be merged based on proximity to one another. Triangulation is chosen opposed to nearest neighbours or distance based on neighbour algorithms because it helps breach the gap between distant areas of the reef. Due to splitting reefs in geomorphic zones, the hexagons are not contiguous, which means that neighbourhood identification methods would result in non-connected graphs (Supplementary Fig. S13).

Each vertex was assigned a weight based on the Euclidean distance for the normalized vertex length and depth difference between the two connected hexagons

$$w_{ij} =\sqrt{v_{ij}^2+(d_i-d_j)^2},$$

where $w_{ij}$ is given as the weight of the vertex between hexagon *i* and *j*, $v_{ij}$ is the length of the vertex between hexagon *i* and *j*, $d_i$ and $d_j$ are the depth of hexagon *i* and *j*, respectively. The minimum spanning tree selects the path along the vertices that minimises the total weight while connecting all hexagons (mst function in the sfnetworks package in R software). This step identifies a path along which hexagons can be merged while minimising the weight, which represents the difference in habitat (Supplementary Fig. S14).

Finally, cluster analysis was used to divide the hexagons into clusters along the path of the minimum spanning tree using SNP-based kinship analysis (skater package in R software). The number of clusters is not fixed and depends on hexagon characteristics (i.e., the homogeneity of depth between hexagons) and the number of hexagons in the network. A minimum size is passed in for a site polygon that represents 200 hexagons which represents 61,400 m^2^, just below the RECOM grid size of approximately 62,500 m^2^.

### Hydrodynamic model

Ocean currents for the Moore Reef cluster were simulated from October to January for 2015, 2016 and 2017 using the SHOC hydrodynamic model^51^ implemented using RECOM^52^. SHOC is a 3D, free-surface, finite-difference, curvilinear-grid, z-coordinate baroclinic model. Bathymetry was interpolated onto the RECOM grid from the GBR100 bathymetry^53^, the grid resolution ranged from 200 to 250 m and vertical resolution ranged from 0.5 m at the surface to 85.5 m in the deepest layers. Atmospheric forcing was obtained from BoM ACCESS Surface Australian Regional Model (ACCESS-R – http://www.bom.gov.au/nwp/doc/access/NWPData.shtml) and wave forcing was taken from Simulating WAves Nearshore (SWAN) as implemented in RECOM. Hydrodynamic initialisation and boundary were taken from the 4 km resolution eReefs hydrodynamic model (version 2.0, https://researchdata.edu.au/ereefs-gbr4-hydrodynamics-v20/681188) using default RECOM boundary settings. Bottom friction in the model was implemented by combining linear and quadratic drag law. The bottom stress ($\tau$) is calculated as

$$\tau = \rho C_d U\text { }max(|U|,U_f),$$

where $\rho$ is water density, $C_d$ is the bottom drag coefficient, *U* is the bottom velocity and $U_f$ is a small background friction velocity, below which the friction law changes from quadratic to linear. $C_d$ is defined as

$$C_d = max\Big (\Big [\frac{1}{\kappa }\ln \Big (\frac{z + z_0}{z_0}\Big )\Big ]^{-2}, C_{d\text { }min}\Big ),$$

where $\kappa$ is the Von Karman’s constant (0.4), *z* is the distance above the sea bed – calculated as the height of the nearest grid point above the bottom, $z_0$ is the bottom roughness point (which may vary spatially) and $C_{d\text { }min}$ is a minimum drag coefficient, typically between 0.002 and 0.003, which places a lower limit on the value of $C_d$ when the nearest grid point is a long way from the bottom (see Herzfeld^51^ for more information).

The hydrodynamic model has been validated at a whole-of-GBR scale using observations from tide gauges, Argo floats, Integrated Marine Observing Systems (IMOS) moorings, Waverider buoys, Australian Institute of Marine Science (AIMS) fixed temperature instruments on coral reef sites, and AIMS water quality moorings^54–57^, but it has not been validated at the scale of this study at this location.

**Figure 9:**
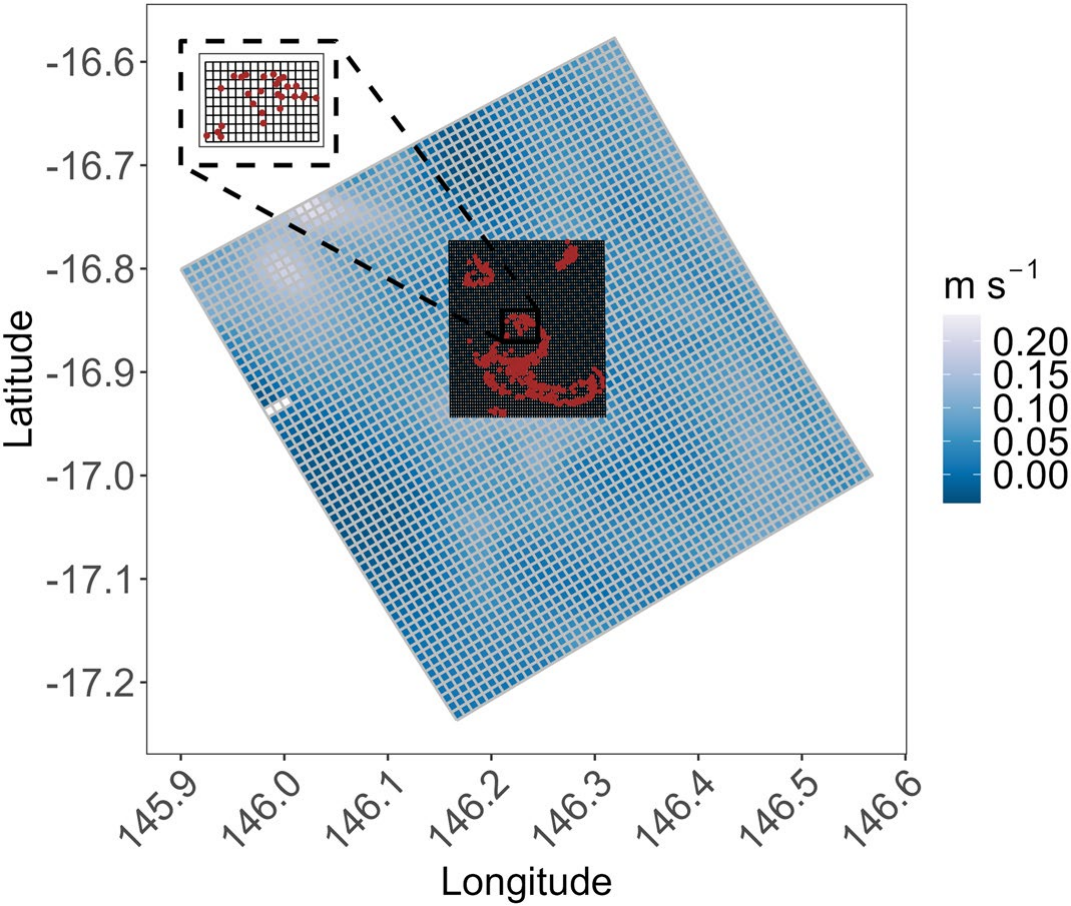
Map showing the Moore Reef Cluster domain obtained from RECOM with all the particle locations (brown) at the time of release nested within a subset of the eReefs GBR1 domain. The grid lines for Moore Reef Cluster correspond to the black lines (200–250 m resolution), whereas the gray lines represent GBR1 grid lines (1 km resolution).

To allow tracking of particles that left the boundary of the RECOM grid during the simulation but later returned (e.g., due to tidal currents or changing wind directions), the RECOM grid was nested within a subset of a larger grid taken from the 1 km resolution eReefs hydrodynamic model, also implemented in SHOC^52^ with 44 depth layers. 3D velocity outputs from the eReefs hydrodynamic model configured at 1 km grid resolution (GBR1) and averaged over a 1.2 s barotropic time step were used with RECOM velocities to create nested velocity fields (Fig. 9). Particles that strayed out of the GBR1 domain (shown in Fig. 9) were removed from the simulation. Both RECOM and GBR1 have curvilinear orthogonal grids. The subset of the GBR1 grid used for nesting consists of 56 cells in the offshore direction, 56 cells in the alongshore direction, and 32 depth levels (deepest layer at 1115 m) with 1 m resolution at the surface. GBR1 forcing was obtained from the same sources used by RECOM for atmospheric and boundary forcing, however, wave forcing was taken from BoM’s regional wave model AUSWAVE-R (configured from WAVEWATCH III at 0.1^∘^) and river flows from 22 rivers. GBR1 velocities used in this study are publicly available in https://dapds00.nci.org.au/thredds/catalog/fx3/gbr1_2.0/catalog.html. Both RECOM and GBR1 represent the influence of wind, waves and tides.

### Particle-tracking model

OceanParcels is a Lagrangian ocean analysis tool that can be used to create customisable particle tracking simulations using velocity outputs from hydrodynamic models. The OceanParcels version^37^ used in this study can read distinct discretised fields, from z- to s- levels in the vertical direction and from rectilinear to curvilinear grids in the horizontal direction. Additionally, it can read distributions in Arakawa’s A-, B-, and C-grids.

### Biophysical model setup

Larval dispersal was simulated for *Acropora* corals because they are abundant on the GBR and provide habitat for many reef associated organisms^58^, are among the most susceptible to coral bleaching and cyclone disturbances, are often a focus for interventions and restoration activities, and because most studies of coral spawning and larval ecology have focused on species of *Acropora* e.g.,^59^. Larval production, dispersal and settlement were simulated for broadcast spawning *Acropora* corals in the Moore Reef cluster of reefs during the annual mass-spawning events in 2015 and 2016, and split-spawning event in 2017. The high RECOM resolution captured small-scale features (e.g., reef wake eddies) which may influence larval dispersal^30,33^. The sensitivity of the biophysical model to the dimension of velocity fields during dispersal were assessed. Utilising 3D RECOM and GBR1 velocities helped resolve the structure of vertical flows within deep waters.

Coral mass spawning by *Acropora* corals usually occurs around the spring-summer transition and peaks 4–6 days after the full moon, but varies depending on the reef location, the timing of the full moon and other environmental conditions^43,44,47^. The spawning days used in this study are as follows: 2015 - November 30, December 1 and 2; 2016 - November 18, 19 and 20; 2017 - November 8, 9 and 10, December 8, 9 and 10 (See Supplementary Information^35^ for more information). Spawning was likely split over two consecutive months in 2017, but with each occurring at a similar time around the full moon and tidal cycle. On the nights of spawning, virtual larvae were released as passive particles at random locations within each of the 334 small polygons (Fig. 1) every 3 min from 8:00 pm to 11:00 pm at 2.25 m below the surface during each spawning day. In each of the 334 spatial polygons, 61 particles were released every spawning day. The total number of virtual larvae released within the cluster domain every spawning day is 20,374.

The particle tracking model was run for a duration of 30 days with a 5-min time step. The released particles were tracked over time and their location was updated every 15 min until the end of the simulation. Although velocities were calculated on a grid resolution of approximately 250 m, the Lagrangian particle tracking on a 15-min timescale allowed tracking of particles on the smaller scale of polygons.

### Post-processing

Larvae were assumed to be neutrally buoyant and competent to settle from 4 to 28 days after spawning^29,36,39^. Larvae were assumed to settle instantly on the first reef polygon within a suitable habitat during the competency period and larvae tracking ceased after settlement was achieved. Since the mortality rate of coral larvae are not precisely known, following Grimaldi et al.^21^, we assume a 0.40 d^-1^ mortality rate by randomly removing 40% of virtual larvae from the total number of larvae each day of dispersion. The information obtained on larvae settlement was used to compute a connectivity (or transfer probability) matrix among sites (or polygons) for each night of spawning.

The proportion of larvae released at a source site that settle on a settling (sink) site was used to calculate the connection strength between source-sink pairs. These connections were computed as links (edges) in the reef network (nodes)^40^.

The sum of each row element is $\le$ 1 because it represents the sum of the proportion of larvae released at a site that settle on one or more other reef sites. Conversely, the sum of each column element can be > 1 since it represents the sum of the proportion of larvae from one or more sites that settle on a particular site.

### Identification of consistent source and settling sites

Reef sites that consistently act as sources and sinks across all spawning days are defined as robust source and sink sites. A source site is consistent if the total proportion of larvae released at the site that settle on one or more reef sites is $>0$ across all spawning nights in the three spawning years. Likewise, a consistent sink site is a site for which the total proportion of the total released larvae that settle on the reef site is $>0$ across all spawning nights in the three spawning years.

### Supplementary Information

Supplementary Figures.

## Data Availability

GBR1 hydrodynamic models developed by eReefs and provided by CSIRO is publicly available at https://dapds00.nci.org.au/thredds/catalog/fx3/gbr1_2.0/catalog.html. Additional RECOM and OceanParcels outputs used in the analyses for this study are available at https://zenodo.org/doi/10.5281/zenodo.10637706.

## Code availability

The code used for analyses is available at https://zenodo.org/doi/10.5281/zenodo.10638015.
