# Supplementary material for: Connectivity modelling identifies sources and sinks of coral recruitment within reef clusters
Source: Sci Rep. 2024 Jun 12;14:13564. doi: 10.1038/s41598-024-64388-8 (PMC11169499; doi:10.1038/s41598-024-64388-8)
Supplement: Supplementary file 1 — Supplementary Figures. [file 41598_2024_64388_MOESM1_ESM.pdf]

Supplementary Information for:

Ani et al. "Connectivity modelling identifies sources and sinks of coral recruitment within reef clusters"

(a) Surface.

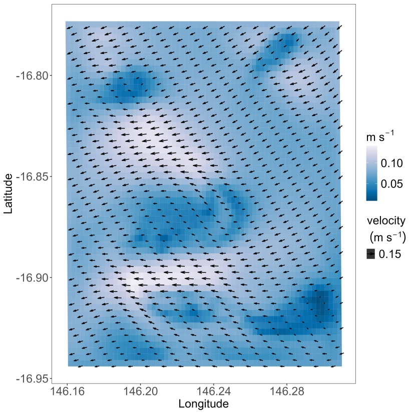

(b) Depth-averaged.

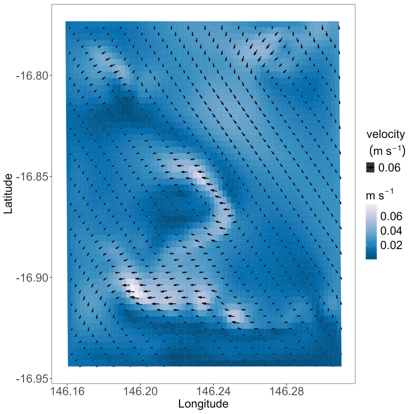

(c) Bottom.

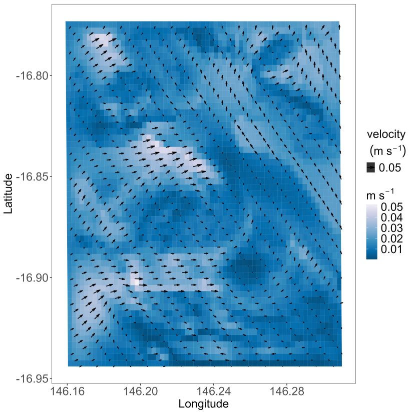

Supplementary Figure S1: Time-averaged surface (a), depth-averaged (b) and bottom (c) flow patterns during the larval dispersal period for 2015 spawning event.

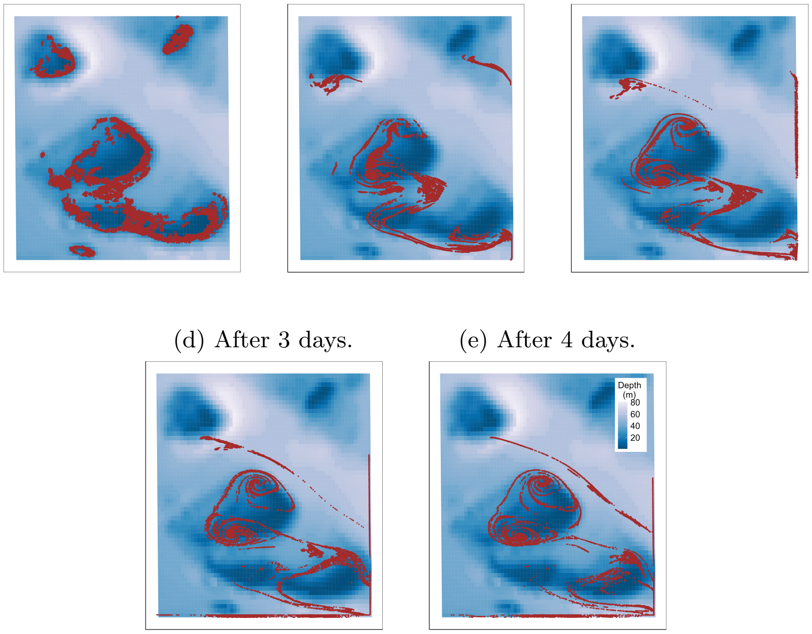

(a) Initial positions. (b) After 1 day. (c) After 2 days.

(d) After 3 days. (e) After 4 days.

Supplementary Figure S2: Snapshots showing the dispersal of *Acropora* coral larvae within the Moore Reef cluster for spawning day 3 in 2015. 2D depth-averaged velocity fields were used to simulate larval dispersal. Brown dots represent particles and the colour bar represents the bathymetry of the cluster. The initial positions of the particles are shown in a, whilst particle positions after 1, 2, 3 and 4 days are shown in b, c, d and e.

(a) Initial positions.

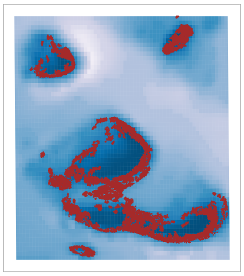

(b) After 1 day.

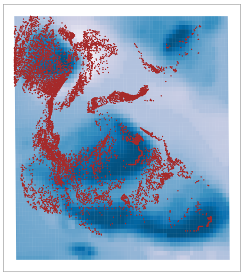

(c) After 2 days.

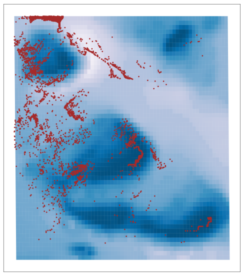

(d) After 3 days.

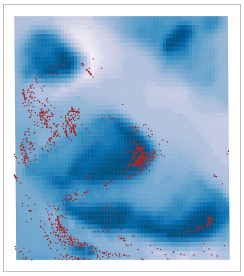

(e) After 4 days.

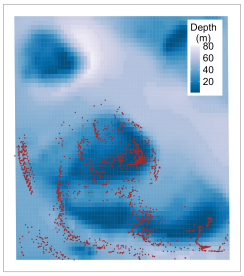

Supplementary Figure S3: Snapshots showing the dispersal of *Acropora* coral larvae within the Moore Reef cluster for spawning day 3 in 2016. 2D surface velocity fields were used to simulate larval dispersal. Brown dots represent particles and the colour bar represents the bathymetry of the cluster. The initial positions of the particles are shown in a, whilst particle positions after 1, 2, 3 and 4 days are shown in b, c, d and e.

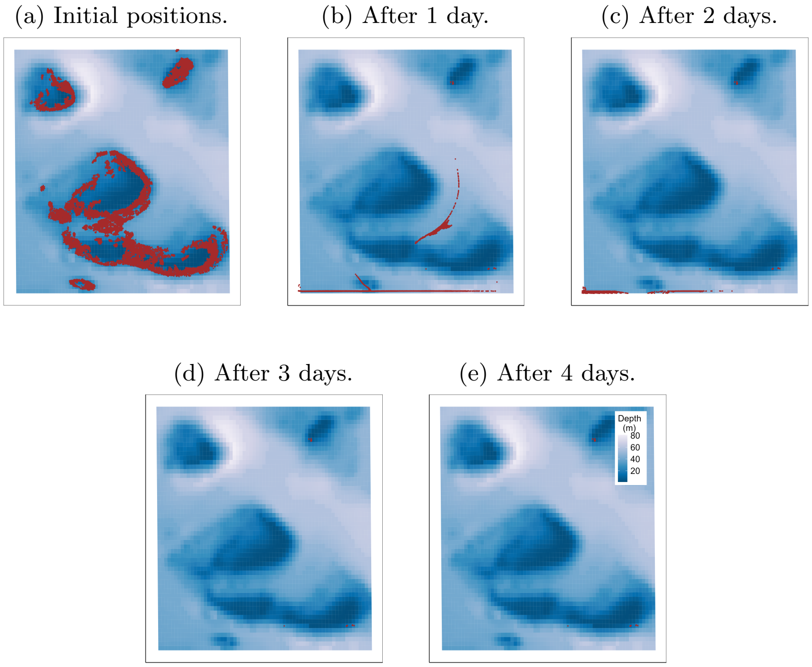

Supplementary Figure S4: Snapshots showing the dispersal of *Acropora* coral larvae within the Moore Reef cluster for spawning day 3 in 2016. 2D depth-averaged velocity fields were used to simulate larval dispersal. Brown dots represent particles and the colour bar represents the bathymetry of the cluster. The initial positions of the particles are shown in a, whilst particle positions after 1, 2, 3 and 4 days are shown in b, c, d and e.

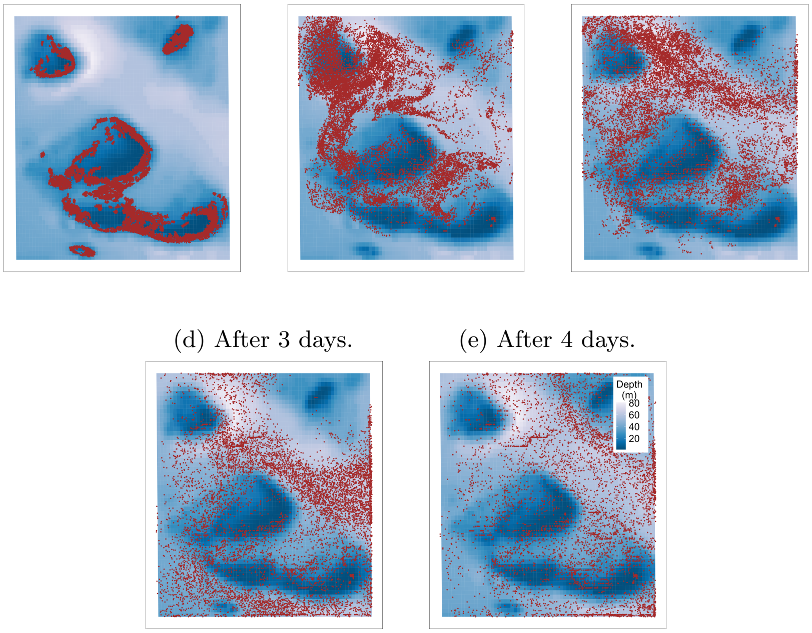

(a) Initial positions. (b) After 1 day. (c) After 2 days.

(d) After 3 days. (e) After 4 days.

Supplementary Figure S5: Snapshots showing the dispersal of *Acropora* coral larvae within the Moore Reef cluster for spawning day 3 in 2016. 3D velocity fields were used to simulate larval dispersal. Brown dots represent particles and the colour bar represents the bathymetry of the cluster. The initial positions of the particles are shown in a, whilst particle positions after 1, 2, 3 and 4 days are shown in b, c, d and e.

(a) Initial positions.

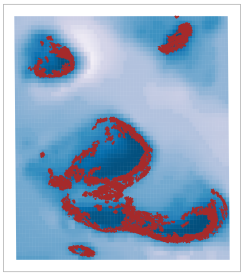

(b) After 1 day.

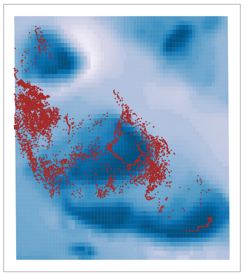

(c) After 2 days.

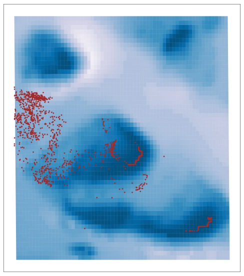

(d) After 3 days.

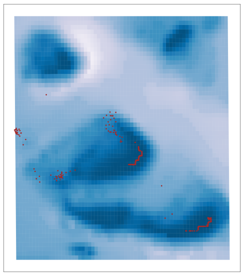

(e) After 4 days.

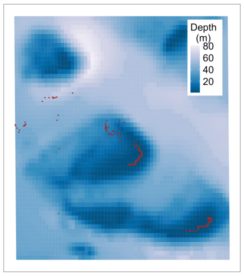

Supplementary Figure S6: Snapshots showing the dispersal of *Acropora* coral larvae within the Moore Reef cluster for spawning day 3 in 2017 first spawning event. 2D surface velocity fields were used to simulate larval dispersal. Brown dots represent particles and the colour bar represents the bathymetry of the cluster. The initial positions of the particles are shown in a, whilst particle positions after 1, 2, 3 and 4 days are shown in b, c, d and e.

(a) Initial positions. (b) After 1 day. (c) After 2 days.

(d) After 3 days. (e) After 4 days.

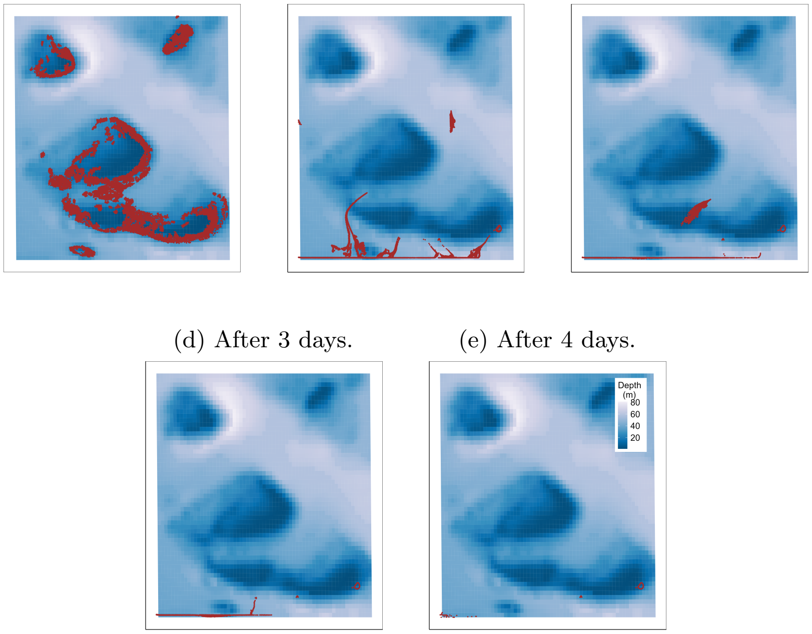

Supplementary Figure S7: Snapshots showing the dispersal of *Acropora* coral larvae within the Moore Reef cluster for spawning day 3 in 2017 first spawning event. 2D depth-averaged velocity fields were used to simulate larval dispersal. Brown dots represent particles and the colour bar represents the bathymetry of the cluster. The initial positions of the particles are shown in a, whilst particle positions after 1, 2, 3 and 4 days are shown in b, c, d and e.

(a) Initial positions.

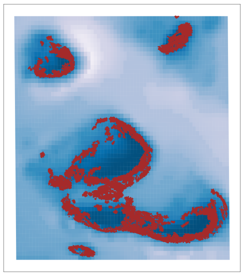

(b) After 1 day.

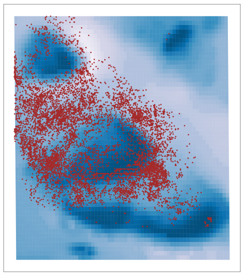

(c) After 2 days.

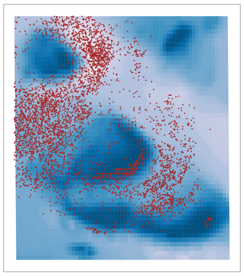

(d) After 3 days.

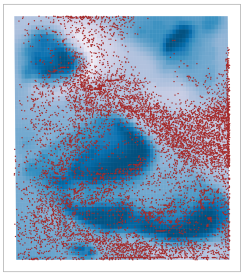

(e) After 4 days.

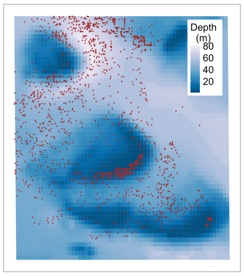

Supplementary Figure S8: Snapshots showing the dispersal of *Acropora* coral larvae within the Moore Reef cluster for spawning day 3 in 2017 first spawning event. 3D velocity fields were used to simulate larval dispersal. Brown dots represent particles and the colour bar represents the bathymetry of the cluster. The initial positions of the particles are shown in a, whilst particle positions after 1, 2, 3 and 4 days are shown in b, c, d and e.

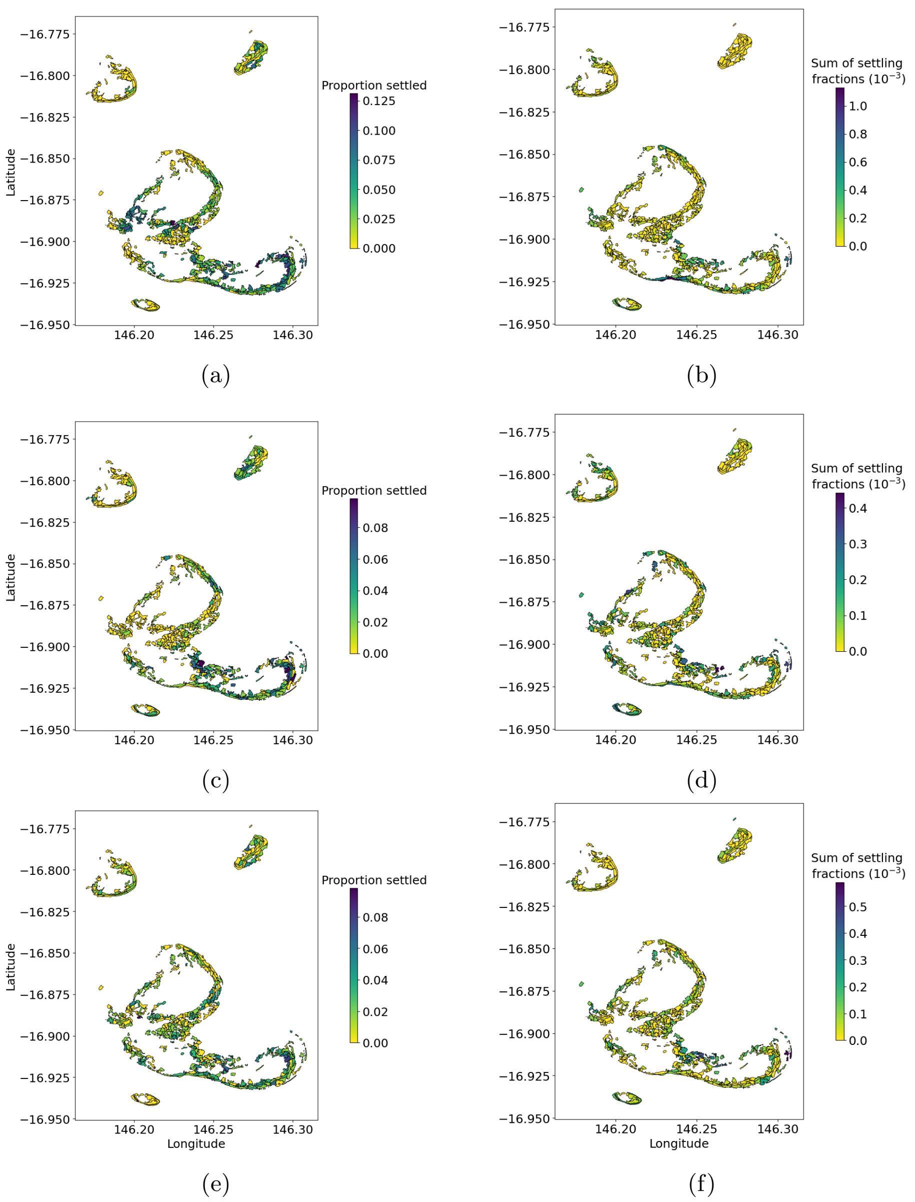

Supplementary Figure S9: Map showing reef sites that act as local sources (a, c and e) and sinks (b, d and f) during the 2016 annual mass spawning. Spawning night 1 (a and b), spawning night 2 (c and d), spawning night 3 (e and f). The colour bar labelled ‘Proportion settled’ corresponds to the proportion of released larvae from each reef site that settle on one or more other sites (a, c and e). The colour bar labelled ‘Sum of settling fractions’ corresponds to the sum of the proportion of the total released larvae from all sites that settle on a particular site (b, d and f).

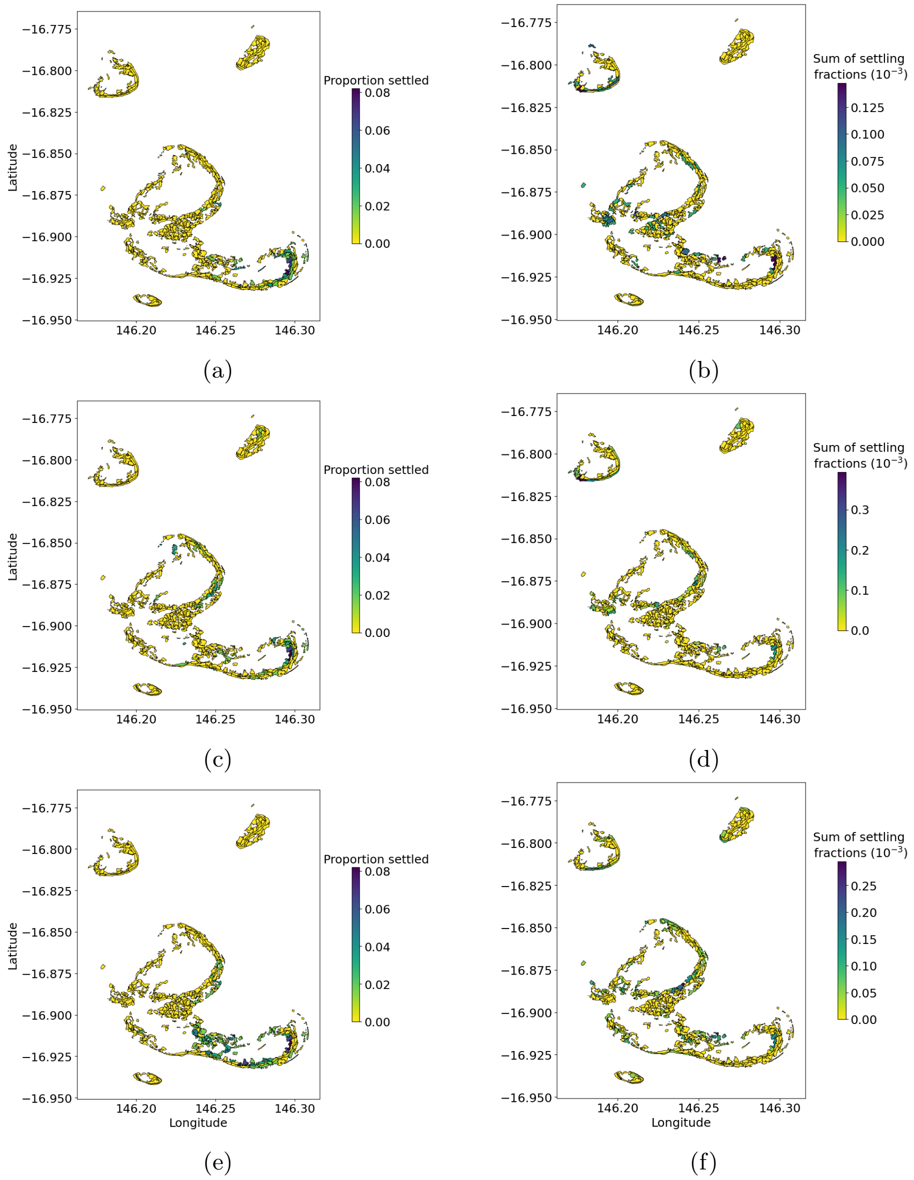

Supplementary Figure S10: Map showing reef sites that act as local sources (a, c and e) and sinks (b, d and f) during the first spawning event in 2017. Spawning night 1 (a and b), spawning night 2 (c and d), spawning night 3 (e and f). The colour bar labelled 'Proportion settled' corresponds to proportion of released larvae from each site that settle on one or more other sites (a, c and e). The colour bar labelled 'Sum of settling fractions' corresponds to the sum of the proportion of the total released larvae from all sites that settle on a particular site (b, d and f).

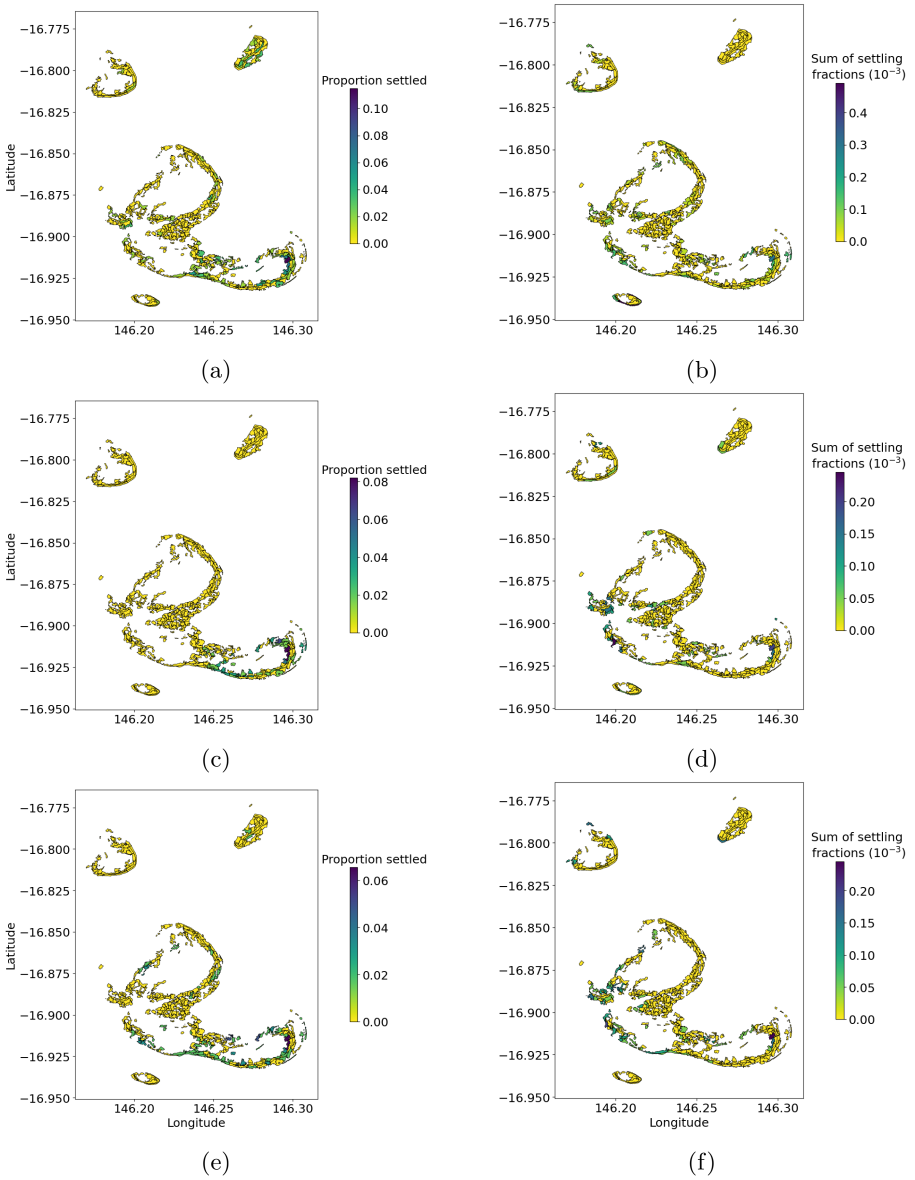

Supplementary Figure S11: Map showing reef sites that act as local sources (a, c and e) and sinks (b, d and f) during the second spawning event in 2017. Spawning night 1 (a and b), spawning night 2 (c and d), spawning night 3 (e and f). The colour bar labelled ‘Proportion settled’ corresponds to the proportion of released larvae from each reef site that settle on one or more other sites (a, c and e). The colour bar labelled ‘Sum of settling fractions’ corresponds to the sum of the proportion of the total released larvae from all sites that settle on a particular site (b, d and f).

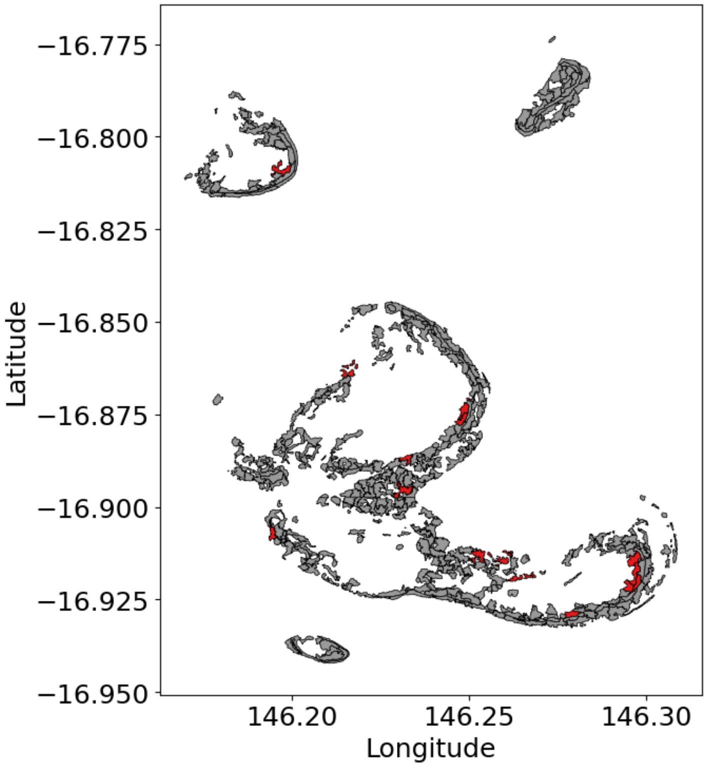

Supplementary Figure S12: Highlighted in red are self-recruiting sites during one or more spawning days.

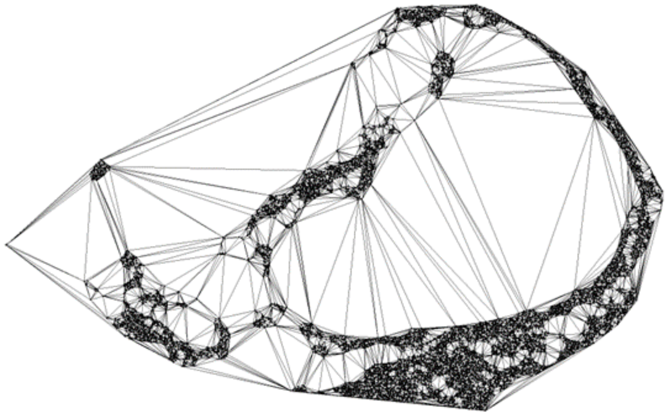

Supplementary Figure S13: Map outlining the Moore Reef cluster including the centroids (black dots) and all possible vertices (grey lines) identified through Delaunay triangulation.

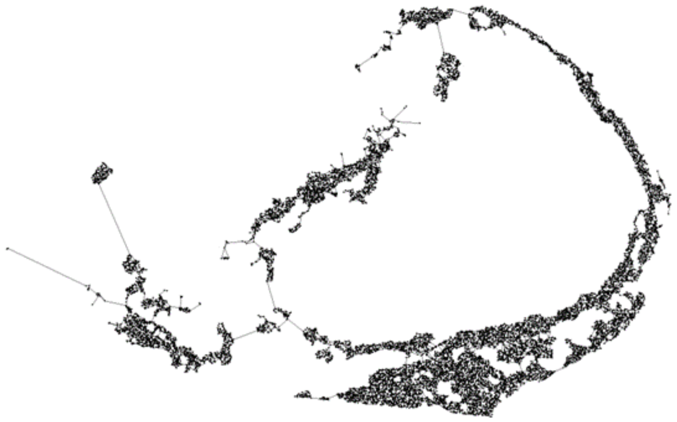

Supplementary Figure S14: Map outlining the Moore Reef cluster including the centroids (black dots) and vertices (grey lines) remaining after the minimum spanning tree was applied.
